# Use of the Versatility of Fungal Metabolism to Meet Modern Demands for Healthy Aging, Functional Foods, and Sustainability

**DOI:** 10.3390/jof6040223

**Published:** 2020-10-15

**Authors:** Jacqueline A. Takahashi, Bianca V. R. Barbosa, Bruna de A. Martins, Christiano P. Guirlanda, Marília A. F. Moura

**Affiliations:** 1 Department of Chemistry, Exact Sciences Institute, Universidade Federal de Minas Gerais, Pres. Antônio Carlos Avenue, 6627, Pampulha, Belo Horizonte 31270-901, MG, Brazil; biancavrb99@gmail.com (B.V.R.B.); bruna.almeidamartins@gmail.com (B.d.A.M.); 2 Department of Food Science, Faculty of Pharmacy, Universidade Federal de Minas Gerais, Pres. Antônio Carlos Avenue, 6627, Pampulha, Belo Horizonte 31270-901, MG, Brazil; cpguirlanda@gmail.com (C.P.G.); mourafmari@gmail.com (M.A.F.M.)

**Keywords:** fungi, secondary metabolites, metabolomics, NTCD, additives, functional foods, nutraceuticals, sustainability, healthy aging

## Abstract

Aging-associated, non-transmissible chronic diseases (NTCD) such as cancer, dyslipidemia, and neurodegenerative disorders have been challenged through several strategies including the consumption of healthy foods and the development of new drugs for existing diseases. Consumer health consciousness is guiding market trends toward the development of additives and nutraceutical products of natural origin. Fungi produce several metabolites with bioactivity against NTCD as well as pigments, dyes, antioxidants, polysaccharides, and enzymes that can be explored as substitutes for synthetic food additives. Research in this area has increased the yields of metabolites for industrial applications through improving fermentation conditions, application of metabolic engineering techniques, and fungal genetic manipulation. Several modern hyphenated techniques have impressively increased the rate of research in this area, enabling the analysis of a large number of species and fermentative conditions. This review thus focuses on summarizing the nutritional, pharmacological, and economic importance of fungi and their metabolites resulting from applications in the aforementioned areas, examples of modern techniques for optimizing the production of fungi and their metabolites, and methodologies for the identification and analysis of these compounds.

## 1. Health and Modern Food Demands

Health concerns have always existed among humans. Although some conditions and diseases cannot be avoided yet, the manifestation of several non-transmissible chronic diseases (NTCD) with high prevalence in patients over 60 years of age such as diabetes, cardiovascular, and neurodegenerative diseases can be delayed by adhering to a healthy lifestyle, which among other factors, is directly correlated to eating habits. The physiological effects associated with the consumption of certain foods are thus becoming very popular. Several types of diets and foods such as the fat-free diet, low-carb diet [1], Mediterranean diet [2], and soy-based diet [3] have been adopted in the quest for healthy aging. Several effects of NTCD have also been postponed through calorie restriction diets in animal models [4]. However, prolonged caloric restriction in humans generates undesirable effects; thus, alternative ways of preventing NTCD have been sought through the development of drugs, foods, and/or nutraceuticals that have both health-promoting and anti-aging effects, without causing adverse effects [5]. There is an increasing trend to combine the use of nutraceuticals with pharmacotherapy, even among individuals with non-aging-related diseases.

As oxidative stress is among the metabolic factors and pathways most related to cell aging, the consumption of nutraceuticals and functional foods with antioxidant activity has increased. Antioxidants can benefit the human body by directly or indirectly neutralizing reactive oxygen species (ROS), modulating metabolic pathways and gene expression, and activating mechanisms of cellular stress and autophagy that delay aging through pathways unrelated to ROS [5]. The nutraceutical market has already reached market values up to USD 117 billion [6]. Nutraceuticals can be classified into several categories based on the level of innovation and area of application (Table 1) [7,8]. Another trend associated with health improvement and NTCD prevention is that of consuming natural foods or foods containing natural, rather than synthetic, additives such as natural flavoring agents, acidulants, and colorants. Several people also prefer vegetarian and vegan diets, which involve restrictions in food and additives of animal origin to different extents; such diets are mainly motivated by the environmental impacts of livestock farming and animal welfare and have prompted studies on the possible effects of these diets on health [9] and increased the market demand for new vegetarian and vegan food products.

Filamentous fungi are capable of responding to different demands for the development of functional foods, nutraceuticals, and bioactive substances that can be used as medicines or in the food industry, either through the use of their biomass or the metabolites produced by them. A comprehensive review of the various aspects of fungal biotechnology and industrial applications was recently published by Meyer et al. [10].

**Figure 1:**
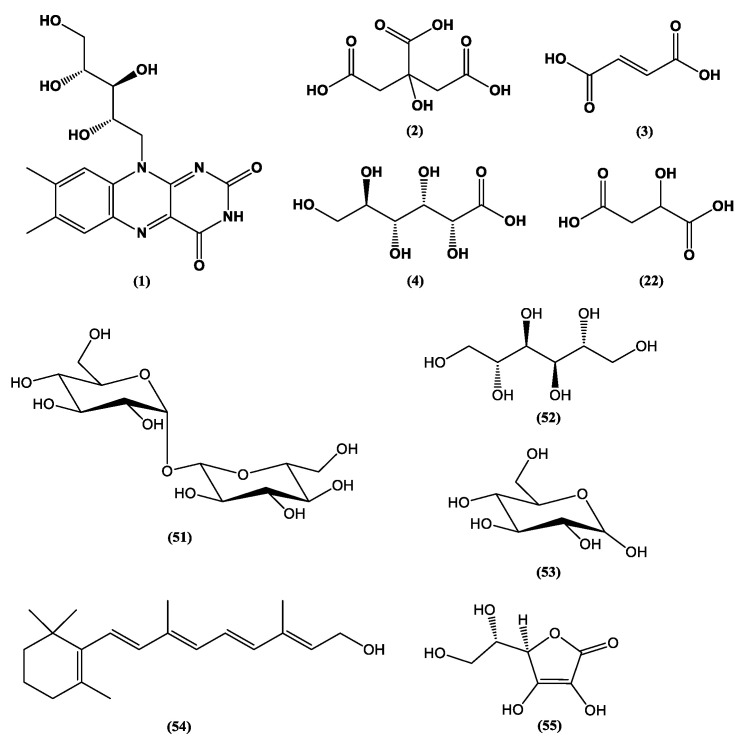
Chemical structures of some fungal-derived food additives **(1–4, 22, 51–55)**.

The consumption of fungi as food mainly involves the consumption of mushrooms (Ascomycota and Basidiomycota phyla), which are enjoyed worldwide, sometimes as delicatessen or gourmet products. However, the role of fungi in human and animal health extends much further than the recognized health benefits of mushrooms [11]. Many fungal species are commercially available as supplements or nutraceuticals, and the fungal metabolites produced by these organisms including many non-Basidiomycota species as functional foods have multiple pharmacological activities. Examples of fungal species used as functional foods include many species of microscopic filamentous fungi that are easily cultivated under ex situ scalable conditions such as some well-known *Penicillium*, *Aspergillus*, and *Fusarium* species. However, lesser known species such as *Ashbya gossypii* also play an important role in the production of food additives such as riboflavin (vitamin B2) **(1)** (Figure 1) [12]. Other important fungal products associated with health improvement are enzymes such as β-galactosidase, which hydrolyzes lactose from dairy foods, is produced by filamentous fungi such as *Trichoderma* sp., and is helpful for lactose-intolerant individuals [13].

This review discusses the usefulness of metabolites produced by fungi for food and pharmaceutical purposes closely linked to health improvement and the prevention of NTCD, respectively, as lead compounds, additives, nutraceuticals, supplements, and functional ingredients. The health benefits of fungal metabolites are highlighted. Cutting-edge tools for yield improvement and thus the scaling up of fungal metabolite production as well as the means for ensuring a successful circular economy in this area are also discussed.

## 2. Natural Food Additives from Fungi

Prompted by the growing evidence for the association between natural compound intake and health, there is a demand for the replacement of synthetic food additives with natural products. Fungal metabolites feature many properties that have been explored for such replacement. This section presents the potential of fungal metabolites as food additives, focusing on their versatility as coloring agents, producing species, and recent achievements and challenges in this area.

Fungi provide several food additives and technology adjuvants such as organic acids, colorants, and fatty acids including some ω-3 and ω-6 class fatty acids, which are essential for human metabolism. Citric acid **(2)** and fumaric acid **(3)** (Figure 1), metabolites of *Aspergillus niger* and *Rhizopus oryzae*, respectively, are outstanding food additives that are industrially produced. Citric acid **(2)** and gluconic acid **(4)** (Figure 1) are fungal products with the highest commercial-scale production [24]. Citric acid **(2)** has a number of applications as an antioxidant, preservative, acidulant, pH control agent, and flavor regulation agent, with market numbers predicted to reach USD 3.6 billion in 2020 [24].

The harmful effects of synthetic food colorants on human health such as attention-deficit/hyperactivity disorder, asthma and allergies, cancer, and neurological disorders have accelerated the search for natural substitutes. The use of the yellow coloring compounds tartrazine, quinoline, and sunset as well as the red coloring compound amaranth [25] has been regulated and supervised by the World Health Organization. In addition, sustainability issues have contributed to the decreased acceptability of non-biodegradable synthetic colorants that are difficult to remove from effluents, causing toxic effects on plants, bacteria, algae, fishes, and crustaceans [26]. Fungal pigments and dyes have emerged as alternatives to these synthetic food additives [27].

**Figure 2:**
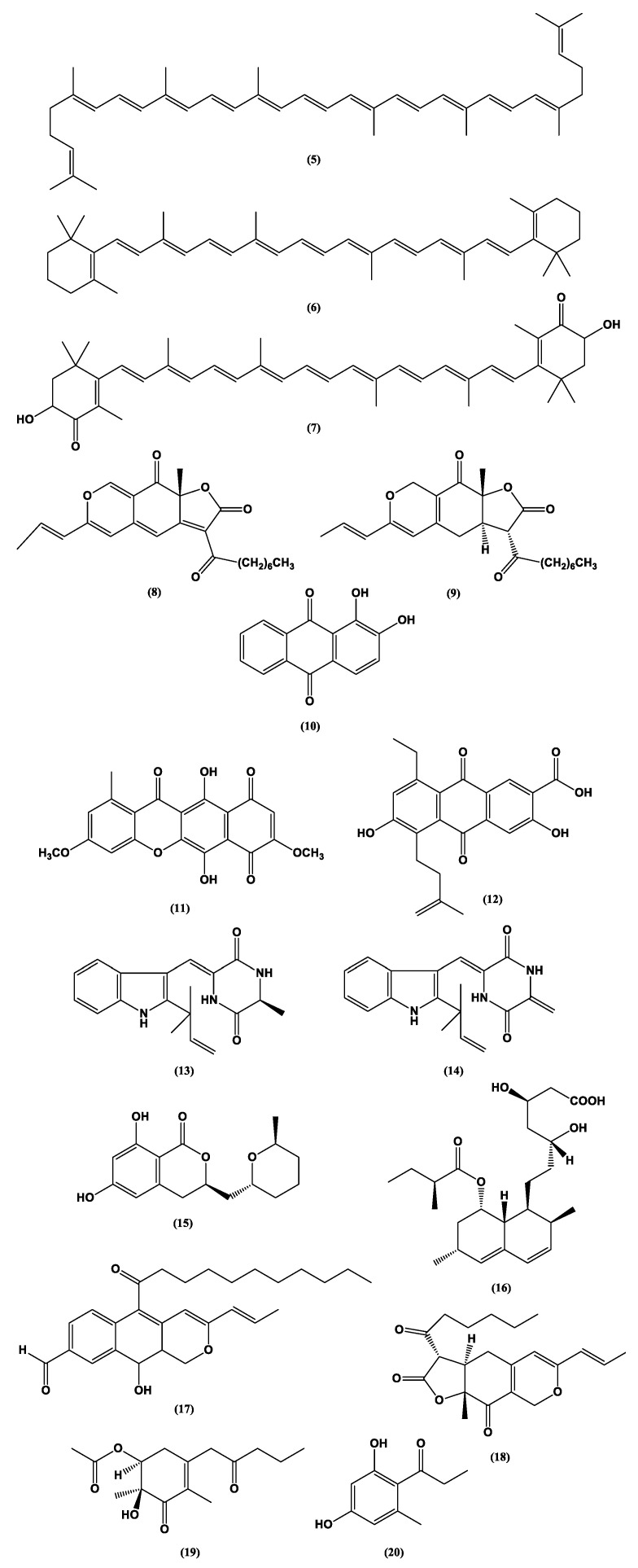
Chemical structures of some colored fungal secondary metabolites **(5–20)**.

Food dyes are coloring materials soluble in food substrates, while food pigments are insoluble in food and need to be carried by vehicles that bind to the food instead [28] (ACS, 2020). Food colorants are pigments or dyes approved for use as food additives [29] (FDA, 2017). A wide spectrum of natural colors can be obtained from metabolites of fungi of different genera such as *Eurotium*, *Fusarium*, *Monascus*, and *Penicillium*, isolated from marine and terrestrial environments and extracted by techniques considered environmentally friendly including those employing ultrasound, pressurized liquid, microwaves, and pulsed electric field [30]. The fungal metabolites have a wide color range, which can be represented by carotenoids such as lycopene (red) **(5)**, β-carotene (yellow-orange) **(6)**, and astaxanthin (pink-red) **(7)**; azafilones such as monascorubrin (orange) **(8)** and ankaflavin (yellow) **(9)**; and the quinine derivatives alizarin (purple-red) **(10)** and bikaverin (red) **(11)** (Figure 2). Arpink Red **(12)** (Figure 2), an anthraquinone produced by *Penicillium oxalicum*, has been approved by the Codex Alimentarius for use as a food colorant in meat products, dairy, confectionery, ice cream, and alcoholic and non-alcoholic beverages [31,32]. Naftoquinone hydrosoluble metabolites (purple color) were produced by a soil-originated strain of *Fusarium oxysporum* using a simple culture medium containing glucose, ammonium sulfate, and salts [33]. Crystalline neoechinulin A **(13)** (ivory color), neoechinulin B **(14)**, and cladosporin **(15)** (all having a yellow color) (Figure 2) are described as metabolites of *Eurotium amstelodami*, *Eurotium herbariorum*, and *Eurotium rubrum* isolated from outdoor and indoor samples in Canada and grown in medium containing sucrose, yeast extract, and salts [34].

The filamentous fungus *Monascus purpureus* is one of the first species that was used to produce natural colorants. It is traditionally consumed in Asia in fermented red rice, which is important in Chinese cuisine, and used in folk medicine as a regulator of digestive and circulatory functions [35,36]. Some species of the genus *Monascus* produce the secondary metabolite monacolin K **(16)** (Figure 2), which consists of a lactone with a free hydroxyl-acid moiety. Aside fromits coloring property, monacolin K **(16)** has a high antioxidant activity and is marketed as a hypocholesterolemic drug known as lovastatin. The pigments of yellow, orange, and red color produced by the *Monascus* genus are considered safe for human consumption [37].

The development of mutant strains of *Monascus* has enabled an increase in available pigments including monashin **(17)** (Figure 2), which is obtained through the mutation of the enzyme polyketide synthase and has an antioxidant activity [35]. The monascin **(18)** (Figure 2) (yellow) produced by *M. purpureus* after activation by the transcription factors DAF-16/FOXO increased the production of superoxide dismutase and thermal shock protein HSP16.2, improving survival in a worm model [38]. Monascin **(18)** also reduced non-alcoholic fatty liver disease and increased AMPK levels and γ 1α co-activator of the receptor activated by peroxisome proliferator in mice [39]. In another study, a new azaphylone, monapurpureusone **(19)**, and a new brownish natural product, monapurpureusin **(20)** (Figure 2), were obtained from a mutant strain of *M. purpureus* cultivated in fermented rice extract. Both compounds presented superoxide radical scavenging activity (EC_50_ = 176.2 and 271.2 μM, respectively), with monapurpureusone **(19)** superior to the control gallic acid (237.1 μM), and anti-inflammatory activity (IC_50_ = 27.5 and 24.9 μM, respectively), with both compounds superior to the control quercetin (35.9 μM) [36].

Red and orange pigments were produced by a strain of *Talaromyces albobiverticillius* isolated from a marine environment at pH 6.5, and their colors were shown to be dependent on the fermentation period (198.6 and 229.0 h, respectively) [40]. Optimal conditions for the production of pigments were also described for *F. oxysporum* (red color, rate C:N = 9, blue LED light, and absence of co-culture) and *Aspergillus chevalieri* (yellow color, rate C:N = 20, glucose as the carbon source, UV and red light, and co-cultivation with *Kluyveromyces marxianus*) [41]. The production of natural food colorants such as melanins, azaphilones, flavins, phenazines, and quinines by filamentous fungi has also been reported for Basidiomycetes [28].

The replacement of synthetic colorants with fungal pigments and dyes enables the production of safer and healthier foods. Compared with plant sources of natural pigments, fungal sources are more economically attractive, considering the relative ease of yield maximization by manipulating fungal fermentative parameters instead of relying on seasonal factors, as may occur in the production of pigments and dyes of plant origin.

**Figure 3:**
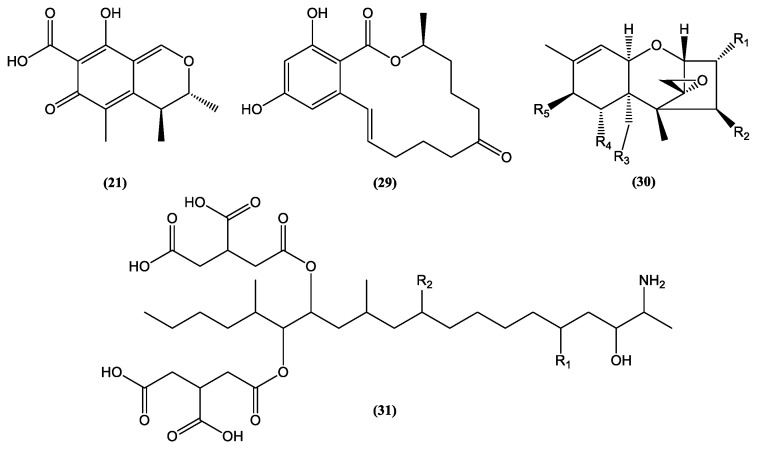
Chemical structures of some mycotoxins **(21, 29)** and classes of mycotoxins **(30, 31)** frequently produced by fungal species.

Nevertheless, some challenges need to be considered for regulating new food-related colorants such as the possibility of interactions with the food matrix, which causes undesirable sensorial changes, loss of color stability, and contamination by toxic substances such as mycotoxins. Some alternatives proposed for these problems are based on the controlled release of the colorant using microencapsulation as well as the use of nanoformulations to eliminate undesirable aromas and flavors [30]. Spray-drying microencapsulation was successfully used in broth fermented by three species producing yellow dyes, *Aspergillus keveii*, *Penicillium flavigenum*, and *Epicoccum nigrum*. Encapsulation with three adjuvants (maltodextrin, modified starch, and gumarabic) provided pigment retention above 70% [41]. With respect to problems linked to the production of mycotoxins such as citrinin **(21)** (Figure 3) produced by *Monascus*, strategies vary from changes in cultivation and fermentation conditions to disruption of genes encoding the production of mycotoxins in question to create non-mycotoxin-producing mutant strains [30].

The use of fungal metabolites as natural colorants contributes greatly to the development of healthier foods. Nonetheless, several other additives of fungal origin such as substances related to taste and pH (acidulants, flavorings, and sweeteners), texture (thickeners and emulsifiers), and increased shelf life (antioxidants and preservatives) have been studied. Some examples can be found in Table 2. The application of some of these additives including exopolysaccharides is not restricted to the food industry. Fungal exopolysaccharides produced by species such as *Phellinus linteus*, *Ganoderma lucidum*, *Fusarium* sp., *Pleurotus* spp., *Inonotus obliquus*, and *Aureobasidium pullulans* have been employed to aid moisture retention in confectionery, increase the viscosity and crystallization of sugar, and as stabilizers, emulsifiers, and thickening agents [42,43]. These compounds are also of great interest to textiles, food, cosmetics, and pharmaceutical industries and are also important in agriculture as preservatives, bioherbicides, and microbicides.

Metabolic engineering of microorganisms has been successfully employed for producing 42 out of the 316 food additives from numerous species of fungi and bacteria currently approved by the European Union [44]. Using this technique, the production of glutamic acid, a metabolite capable of providing “umami” flavor to food, though fermentation with *Corynebacterium glutamicum* was optimized. The production of malic acid **(22)** (Figure 1), an acidulant used in food and beverages, was increased after overexpression of the genes encoding its precursors in the species *S. cerevisiae*, *Aspergillus flavus*, *Aspergillus oryzae,* and *A. niger* [51,52]. Metabolic engineering can also be used to introduce heterologous routes for enzyme production in microorganisms (e.g., to produce enzymes of plant origin) [51]. Changes in the molecular structures and colors of microbial pigments were made using the engineering system CRISPR-Cas9, which cleaves specific microbial DNA sites and introduces changes in one or more target genes [30]. For instance, *Escherichia coli* was used for the production of β-carotene **(6)** through genomic editing to introduce insertions, deletions, and substitutions in regions of the lacZ, galK, and ldhA genes using the CRISPR-Cas9 system [53].

A yeast species, *Yarrowia lipolytica*, was engineered by introducing genes for the production of β-ketolase and β-hydroxylase of seaweed and bacteria origin, increasing the yield of astaxanthin **(7)** (285 ± 19 mg/L; 47% of total carotenoids) [54]. This was a great achievement, given the lower productivity of astaxanthin **(7)** by yeasts like *Xanthophyllomyces dendrorhous* (10.2 mg/L) [55] and microalgae like *Haematococcus pluvialis* (84.8 mg/L) [56] and *Chlorella zofingiensis* (12.5 mg/L) [57]. Astaxanthin **(7)** has a high market value (USD 2500–7000/kg), 5–20 times higher than that of β-carotene **(6)** produced by the microalga *Dunaliella salina* (USD 300–500/kg) [54,58].

Overall, tools such as genetic manipulation based on transcriptomic analysis, induction of mutations, cloning, and insertion of heterologous plasmids into species with well-known genomes have enabled the production of large quantities of metabolites by previously non-producing species [59]. The elucidation and manipulation of the different stages in the transcription and secretion of amylases, xylanases, and cellulases in filamentous fungi has enabled their overexpression [60]; these enzymes are widely used in bakery products to improve the quality of dough through hydrolysis of long-chain carbohydrates and non-starch polysaccharides (cellulose and arabinoxylans).

It was hypothesized that enzymes produced by *A. oryzae* and *A. niger* could cleave the anti-nutritional factors present in flour, in addition to hydrolyzing proteins and carbohydrates into smaller molecules and thus making them more accessible for digestion and releasing phenolic compounds from the matrix. However, the fungi used the amino acids present in the flour for their own metabolism, which negatively affected the protein quality of the final product [61]. A new fermented food product was thus developed using stale bread as a substrate for the fungus *Neurospora intermedia*, which converted 65% of the starch into 21% of protein, in addition to supplementing the final product with minerals and vitamins; although there was a reduction in the amounts of proline, glutamic acid, and phenylalanine, the overall amino acid composition was improved [62].

## 3. Benefits, Research, and Industrial Applications of Fungal Metabolites

The scope of the pharmacological activity of fungal metabolites seems to be as endless as the structural diversity. However, several issues in this area must be addressed such as the extent of in vivo effects. The wide range of beneficial biological effects of fungal metabolites can be related to the prevention and treatment of NTCD, and some such effects have identified several potential compounds for developing new drugs.

**Figure 4:**
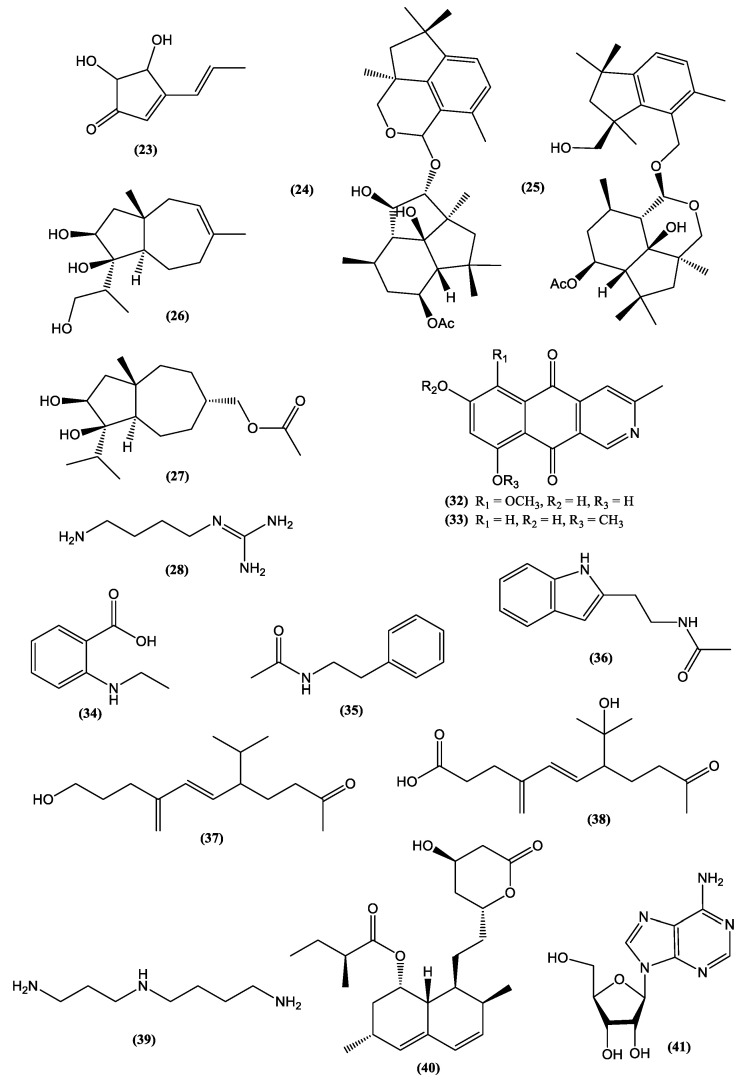

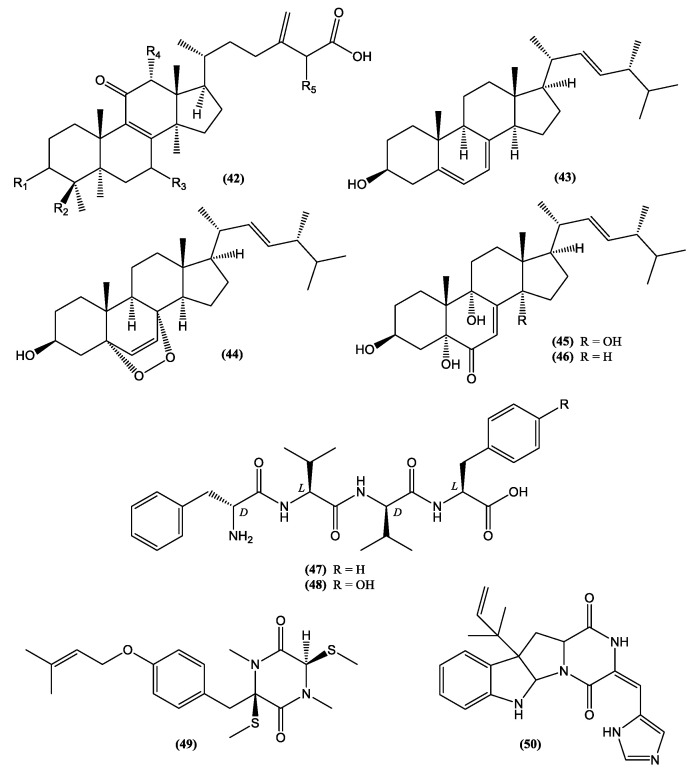
Chemical structures of metabolites **(23–28, 32–41, 43–50)** and class of bioactive compounds **(42)** produced by fungal species.

Terrein **(23)** (Figure 4), for instance, is a secondary metabolite biosynthesized in high concentrations (537.26 ± 23.42 g/kg crude extract) by *A.terreus* [63]. The anti-inflammatory and antioxidant properties of terrein **(23)** were reported in in vitro studies [64]. These important medicinal properties and the high initial yield of this metabolite allow the large-scale production and technological development of the *A. terreus* crude extract for the prevention of some age-related NTCD.

Compounds presenting cytotoxicity against tumor cells form one of the most important classes of fungal metabolites. The world incidence of cancers is high, with cancer affecting one in five people at some point in life. The number of cancer cases registered in 2018 was 18.1 million people, a number that could double in 2040 [65]. Cancer is a multifactorial disease that affects people regardless of age, gender, or origin. As an NTCD, cancer is worrying in several ways, since working-age people can be affected, treatment is costly and long, mortality rates are high, and individuals often have health issues even after cure.

Many reports on fungal metabolites with cytotoxic activity show the vast arsenal of molecules that fungi provide to combat cancer. Table 3 provides examples of antitumor metabolites produced by fungi. Species from different genera or secondary metabolites produced by them sometimes present inhibitory power more pronounced than or relatively close to that presented by standard compounds. This was observed for hypocriol A **(24)** and F **(25)** (Figure 4), isolated from the strain *Hypocrea* sp. [66], and for sesquiterpene strichocaranes E **(26)** and F **(27)** (Figure 4), isolated from the entomopathogenic fungus *Isaria fumosorosea* [67], in comparison to the standard cisplatin. Many human tumor cell lines can be inhibited by fungal metabolites such as breast (MDA and MCF-7) [67], cervical and lung [66], colorectal [63], gastric and liver [68], and pancreatic [69] cell lines. The majority of compounds presented in Figure 4 are terpenes with different degrees of hydroxylation as well as linear and cyclic nitrogen-bearing compounds with free NH groups.

Despite the huge number of fungal bioactive metabolites, there is controversy regarding the consumption or development of new drugs from metabolites biosynthesized by toxin-producing fungi. Terrein **(23)** and agmatine **(28)** (Figure 4)are examples of biologically active metabolites produced by *Aspergillus*, a genus often associated with the production of mycotoxins [71,72]. However, species from *Aspergillus* and *Fusarium* genera, known sources of toxins such as zearalenone **(29)**, trichothecenes **(30)**, and fumonisins **(31)** (Figure 3), have shown a biotechnological potential beyond the production of mycotoxins. For example, azaanthraquinone derivatives, 7-desmethyl-6-methylbostrycoidin **(32)**, and 7-desmethylscorpinone **(33)** (Figure 4), isolated from *F. solani* cultures, showed significant activity against tumor cell lines (Table 3) [69].The mycoprotein “Quorn”, a popular fungal food for human consumption, is produced using mycelia of *Fusarium* sp. [72]. Therefore, disregarding the potential of species from mycotoxin-producing genera is unnecessary in the research and industrial development of food products and medicines.

Cancer and neurodegenerative diseases are pathologies targeted by antioxidant therapies, not only for treatment but also for prevention, as proven by pre-clinical, clinical, and epidemiological studies [73]. Although compounds and foods with antioxidant activity have been targeted by many studies [5,74], some brief considerations are worthwhile. Despite encouraging data from several screenings pointing to a very significant number of fungal products with antioxidant activity, it is important to note that most of the experiments in these studies were not applied to tissues and organ systems, and the pharmacokinetic aspects of the absorption of these substances by the human organism were not evaluated [74]. Thus, the straightforward extrapolation of the results of these screenings to in vivo human applications is not possible [75].

Conversely, the in vitro antioxidant profile of a natural product can be improved. This was observed in a study in which three strains of *M. circinelloides* (CBS 277.49, WJ11, and CBS 108.16) were grown for different time periods (three, five, and seven days) in different culture media (standard Kendrick medium and Ratledge and modified Kendrick and Ratledge media (MKR), with nitrogen deficiency) and evaluated for phenolic compounds and antioxidant capacity. The total phenolics (TPC) and flavonoids (TFC) were improved for CBS 277.49, while CBS 108.16 produced a higher amount of condensed tannins (TCT). The ethanol extract obtained from CBS 277.49 (five days of growth in MKR medium) presented the best results regarding neutralization of the2,2′-Azino-bis(3-ethylbenzothiazoline-6-sulfonic acid)(ABTS^+^) radical, copper reducing power, and ferric reducing power. The latter had a higher ferric reducing power than those of the standards BHT and α-tocopherol [76].

Despite the relevance of cancer as an NTCD, several other diseases linked to aging limit a healthy life as people age. Accumulated dietary deficiencies throughout life and loss of immunity increase mortality rates associated with non-fatal diseases such as infections. Chronic inflammation is another worrisome condition that increases the risk of developing various pathologies such as hypertension, diabetes, and neurodegenerative diseases [77]. Although early diagnosis and measures to avoid aging-related pathologies and their consequences are essential, drugs for existing diseases continue to be required. Table 4 presents the structures of fungal metabolites associated with the treatment or control of infections, inflammatory and neuroinflammatory conditions, and Alzheimer’s disease and examples and extents of the biological activities of these compounds.

The use of epigenetics for the production of new secondary metabolites through the activation of biosynthetic routes involving transcriptional genes previously silenced is an approach to increase and diversify the production of bioactive secondary metabolites by fungi. The use of a mutant strain of *Fusarium graminearum* lacking H3K27 methyltransferase after removal of the secondary metabolism repressor *kmt6* gene was reported as being advantageous for obtaining 22 metabolites, three of which had not been previously identified to be produced by this species: N-ethyl anthranilic acid **(34)**, N-phenetylacetamide **(35)**, and N-acetyltryptamine **(36)** (Figure 4). The production of the double mutant *kmt6fus 1*, with elimination of the production of compounds of the class fusarins, enabled the discovery of two new sesquiterpenes, tricinolone **(37)** and tricinolonic acid **(38)** (Figure 4) [84].

## 4. Mushrooms as Functional Foods for Preventing Aging-Related Non-Transmissible Chronic Diseases (NTCD)

The intake of edible mushrooms as functional foods associated with health improvement has been widely described [85]. Some metabolites obtained from mushrooms have been introduced into the market as they have antitumor properties or immunostimulants such as the polysaccharide lentinan produced by the edible mushrooms *Lentinula edodes* (Shiitake) and *G. lucidum*. *G. lucidum* latter is consumed in traditional East Asian medicine as bitter tea (hot aqueous extraction) and can also be obtained as a dry powder. The pharmacological properties of several mushroom metabolites have been demonstrated such as those of polysaccharides produced by *G. lucidum*, which were able to increase the survival of worms by activating the transcription factors DAF-16/FOXO [86].

*Hirsutella sinensis* produces polysaccharides with prebiotic properties related to insulin resistance and diabetes control [87,88]. Spermidine **(39)** (Figure 4) produced by several species [89], proved capable of prolonging the life of mice by reducing histone acetyltransferase EP300 [90], while epidemiological evidence indicates that spermidine **(39)** intake may also contribute to the reduction of human mortality [91]. Considerable amounts of the metabolite lovastatin **(40)** (Figure 4), a drug registered as low-density lipoprotein (LDL) cholesterol lowering, were detected in the widely consumed mushrooms *Agaricus bisporus* (30.79 mg/100 g dry weight), *Cantharellus cibarius* (67.89 mg/100 g dry weight), *Imleri abadia* (6.21 mg/100 g dry weight), and *L. edodes* (0.95 mg/100 g dry weight) [92].

However, the most prominent example of a mushroom with nutraceutical properties may be *Cordyceps militaris*, a rare and naturally occurring entomopathogenic medicinal mushroom in the Himalayan Mountains, Tibet, Nepal, and India. Studies have reported that consuming *C. militaris* extracts significantly increases glucose metabolism, thereby decreasing the glucose level in the blood. In addition, consumption of this mushroom provides protection against diabetic nephropathy [93]. *C. militaris* produces polysaccharides in the fructification body that are active toward α-glucosidase [94] and have immunomodulatory activity; this suggests their incorporation into functional foods and dietary supplements [95]. Anti-adipogenic activity was reported for a fermentation mix containing strawberry, silkworm pupae, and *C. militaris* [96]. Several nutraceutical products containing *Cordyceps* are available in the global market. Among the benefits claimed by their manufacturers are the promotion of mental health and benefits to the vascular system *(“Cordyceps* active”), cognition support (“Mushroom Plus”), anticancer and antioxidant activity (“Bhutan *Cordyceps* Tea”), strengthening of the cardiovascular system (“MRM *Cordyceps*CS-4 Strain”), and immune system support (“MycoNutri *Cordyceps* Organic”) [97]. Cordycepin **(41)** (Figure 4) is the main metabolite produced by *C. militaris* and is very effective in reducing the accumulation of LDL, total cholesterol, triglycerides, and hyperlipidemia caused by high-fat diets [98]. Cordycepin **(41)** has several pharmacological properties such as anti-inflammatory, immunomodulatory, antioxidant, anti-aging, anticancer, antiviral, cardio, and hepatoprotective properties, among others [97]. This range of activities results in health effects that may help in postponing aging-linked NTCD. *C. militaris* also has high nutritional value and contains proteins, phenolic compounds, steroids, and lectins [99].

Another mushroom species with high functional and nutraceutical potential of high market value is *Agaricus subrufescens* (synonymy *Agaricus blazei* and *Agaricus brasiliensis*) [100]. It is commercialized in several countries such as Brazil (brand name "Sun mushroom"), China (Ji Song Rong), and Japan (Himematsutake) [101]. Sun mushroom contains polyphenols and polysaccharides and is known to decrease oxidative stress and prevent NTCD; it is indicated to have antioxidant, antitumor, anti-inflammatory, and immunomodulatory properties [102].

Another class of bioactive compounds of increasing prominence found in edible mushrooms includes antcins **(42)** (Figure 4), steroids that contain an ergostane-type skeleton and are produced by *Antrodia* species such as *Antrodia cinnamomea* and *Antrodia salmomea*. Studies suggest that these compounds are promising agents in the treatment of cancer, inflammation, diabetes, and diseases resulting from oxidative stress, among others. The aforementioned species have been historically used in communities of Taiwan for treating various diseases such as diarrhea, abdominal pain, hypertension, dermatological irritation, and intoxication by food, alcohol, and drugs [103]. A study tracking 36,499 middle-aged and elderly Japanese men over an average of 13.2 years found a positive relationship between regular mushroom consumption and decreased incidence of prostate cancer [104]. Table 5 summarizes the classes of compounds and health benefits from some mushroom species cited in this section.

## 5. Toward a Sustainable Production of Fungal Metabolites

As the demand for preventive medicines, nutraceuticals, new drugs, food additives, and other health-related products of natural origin grows, the need for scaling up is also increasing. For bioactive metabolites of plant origin, efforts to increase production may be slow because production sometimes dependent on the seasonality of plants and a long plant growth period. Thus, efforts to increase the production of bioactive compounds have been directed toward microorganism-based options, such as metabolic engineering. Modifications in the culturing of fungi have been successful in the yield improvement of biomass and bioactive compounds. Moreover, special consideration is being given to endophytic fungi, especially those able to produce metabolites biosynthesized by their host plants. Table 6 shows some interesting examples of bioactive compounds produced by endophytic fungi after optimization of fermentation conditions. The good outcomes in this area were exemplified by Torres-Mendoza et al. [105] who reported, from 2001 to 2019, 224 patents related to metabolites from endophytic fungi applied to agricultural, biotechnology, pharmaceutical, and food industries, most of which used species from the *Aspergillus*, *Fusarium*, *Trichoderma*, *Penicillium*, and *Phomopsis* genera.

Yield enhancement in microbial production is one of the most challenging issues, but good progress has been reported. The production of fungal metabolites can also be optimized by co-cultivation with other fungal or bacterial species and variation of chemical parameters, such as composition of the culture medium, and physical parameters, such as temperature, pH, stirring speed, intensity and color of light, and oxygenation. The metabolic modulation resulting from these approaches depends on the fungal species; thus different fungi have been explored to produce fungal metabolites with new industrial applications around the world [38].

Biosynthesis of the natural polyamide agmatine **(28)** by *A. oryzae*, a fungus generally recognized as safe, was described during the fermentative process of sake production (Japanese rice wine) [118]. Optimization of the initial yield of agmatine **(28)** produced by *A. oryzae* in the presence of *S. cerevisiae* in a solid state (3.5 mM agmatine) was achieved by varying some fermentative parameters. At pH 5.3, the production of agmatine **(28)** increased to 6.3 mM. An increase of over 100% in the initial productivity was obtained by adding ʟ-lactic (pH 3.0, 8.2 mM agmatine), succinic acid (pH 3.5, 8.7 mM agmatine), and citric acid **(2)** (pH 3.2, 8.3 mM agmatine) in the fermentation medium. Therapeutic evidences indicate that agmatine **(28)** is a promising lead compound against several NTCD that affect the central nervous system, such as Alzheimer’s disease [119]. In 2019 only, the number of people affected by dementia was estimated at 50 million, with the worrying forecast of this number to be trebled by 2050 [120]. Therefore, new molecules for the treatment of Alzheimer’s disease and other types of dementia are highly desired. The pharmacological properties and yield of agmatine **(28)** in fermentation by *A. oryzae* suggest the possibility of its incorporation into nutraceuticals [119]. In excess, agmatine (**28)** may present toxicity and can enhance the toxic action of other biogenic amines, such as histamine and tyramine, produced by the decarboxylation of amino acids by food-fermenting microorganisms. Therefore, determining a proper intake for agmatine **(28)** is necessary to mitigate risks and allows for the numerous aforementioned health benefits [16].

The preference for faster, less expensive, and green approaches has sped up research on bioactive fungal metabolites. Some promising analytical tools, such as matrix-assisted laser desorption ionization time-of-flight mass spectrometry (MALDI-ToF MS), have been useful for addressing some bottleneck problems in this area, such as fungal identification. Genetic and morphological methods, although very efficient, require expertise, time, and resources that may be saved by the use of analytical instrumentation [121]. Such improvements start during screening steps, in the initial laboratory prospection of relevant species and promising fermentative conditions, and continue to the production steps, with the use of alternative substrates for fungal growth, such as agro industrial residues, towards a circular economy. Thus, extracts intended for screening are prepared on a reduced scale, using lower amounts of reagents, such as solvents, during extraction. In addition, modern analytical tools coupled with hyphenated techniques have been recently used for the analysis of a greater number of extracts, without the need for prior isolation of fungal metabolites from extracts. Biochemometrically derived fingerprints obtained by gas chromatography, high-performance liquid chromatography, MS, and nuclear magnetic resonance spectroscopy (NMR), together with statistical analysis also enable the establishment of straightforward associations between metabolite content and biological activities detected for a large number of crude extracts [70,122].

Although MS is the most widely used technique for this kind of analysis [123], NMR, in particular ^1^H NMR metabolomic analysis, has gained ground by enabling the nondestructive identification of molecules in complex mixtures originating from various biological materials, such as plants and fungi extracts, foods, biological fluids, and tissues [124,125]. NMR is comparable to MS in criteria, such as efficiency, speed, reproducibility, and ease of sample preparation; however, it has advantages in terms of sample recovery and isotope detection. It is also independent of ionization potential and does not require complex internal standards [126]. Bi-dimensional biochemometric NMR evaluation of the crude extract of a marine-derived strain of *P. chrysogenum* enabled the attribution of the anti-proliferative activity of the extract against breast cancer cell lines to ergosterol **(43)** (Figure 4; Table 3), a structural metabolite common in fungi [70]. In addition, other metabolites of the ergostane class, ergosterol peroxide **(44)**, 3β, 5α, 9α, 14 α-tetrahydroxy-ergosta-7,22-dien-6-one **(45)**, and 3β, 5α, 9α-trihydroxy-ergosta-7,22-dien-6-one **(46)** (Figure 4), were detected as metabolites of the edible mushroom *V. volvacea* and proved to be active against human cancer cell lines (Table 3) [68].

An interesting application of NMR-based metabolomics for the comparison and origin identification of edible mushrooms was reported. Multivariate analyses, such as PCA, were used to compare the chemical profiles of species, enabling the differentiation of *Kuehneromyces mutabilis* and *Hypholom acapnoides,* and the direct identification of 17 secondary metabolites in their extracts. Statistically significant differences were observed inthe^1^H NMR data in the variation of composition based on the collection site and restrictedness of metabolites in *K. mutabilis*. Upon comparing *H. capnoides* and *K. mutabilis*, a higher diversity of sugars was observed in *H. capnoides*, while *K. mutabilis* presented significantly higher amounts of some organic compounds, such as fumaric acid **(3)**, showing the versatility of the ^1^H NMR technique for comparing fungi in terms of metabolites [127].

Other sophisticated approaches combining UPLC−ESI-TOF/MS, differential off-line LC−NMR, and quantitative^1^H NMR (qHNMR) analysis were used to identify ᴅ-Phe-ʟ-Val-ᴅ-Val-ʟ-Tyr **(47)**, ᴅ-Phe-ʟ-Val-ᴅ-Val-ʟ-Phe **(48)**, and cis-bis(methylthio)silvatin **(49)** (Figure 4) in the aerobic fermentation of *Penicillium roqueforti* with and without L-Tryptophan enrichment. Another metabolite, roquefortine C **(50)** (Figure 4), was identified as an antimicrobial agent against *Bacillus subtilis* and *E. coli* [126]. ^1^H NMR-based metabolomics, applied in a study on extracts of 11 species of edible mushrooms, identified dry *Pleurotus geesteranum* and *Hericium erinaceus* and fresh *Pleurotus sapidus* as the most prominent species in terms of metabolite production. In addition, the use of ^1^H NMR led to the detection of more than 100 different metabolites of interest to the food industry including trehalose **(51)**, mannitol **(52)**, and glucose **(53)** (Figure 1). The analysis also showed high levels of carbohydrates and proteins, in addition to considerable amounts of vitamins A **(54)** and C **(55)** (Figure 1) and amino acids as nutritional compounds [14]. Therefore, NMR-based metabolomics has been successful in selecting fungi with outstanding potential for further technological development. Metabolomic tools have also been applied for the evaluation of organoleptic properties and interference of external factors with food quality [128] and nutrient quantification such as in the determination of the nutritional contents of 11 *Capsicum annuum* cultivars in terms of ascorbic acid (vitamin C) **(55)** [129]. In the near future, once there is sufficient data in this area, these useful metabolomic tools could be applied in several meta-studies such as for the determination of vitamin C **(55)** content in edible mushrooms. One study [130] reported vitamin C contents between 31.16 ± 0.93 (*Calvatia gigantea*) and 108.11 ± 3.22 (*Lepis tagilva*) mg/kg dry matter. In another study [14], the vitamin C contents ranged from 0.5 to 111.4 mg/100g dry weight. The highest levels of vitamin C were produced by the species *D. indusiata* and *A. subrufescens* at 111.4 and 69.7 mg/100 g dry weight, respectively [14], values close to the daily levels of vitamin C **(55)** recommended in some countries (75 to 110 mg) [131].

Metabolomic approaches have also been applied to the quantification of aflatoxins in industrialized baby food [132], to determine adulteration and its effect on the safety of nutraceuticals [133], and for differentiating extracts of various biomaterials such as crops [134] and fungi [14].

While metabolomic tools provide speed for research, technological development for future industrial applications of fungal metabolites must consider that the modern economy is increasingly challenged to transform traditional processes into sustainable production chains, minimizing waste, and reusing biomass for applications in the food and drug industries, even for well-established processes such as citric acid **(2)** production. The global market value of citric acid **(2)** was estimated to increase to USD 3.6 billion in 2020 and maintain an annual growth of 5% until 2025. Despite the successful industrial experience for citric acid **(2)** production, research to increase sustainability in bio-refineries is ongoing, opening new possibilities such as clustering-related fermentation production including olive oil and wine to reach models for circular bioeconomy [135].

Fortunately, the production of nutraceuticals and functional foods from biomass, extracts, and fungal metabolites greatly addresses the challenge of sustainability improvement, allowing the incorporation of low-cost, underutilized, and abundant materials into the industrial fermentation process. Many agro-industrial residues available all over the world are sources of peptides, prebiotic dietary fibers, and hydrolyzed or smaller organic molecules such as phenolics, carotenoids, and tocopherols, among other classes. These features make them good materials for fungal cultivation as well as interesting substrates for the production of bioactive components from macromolecules [59,136]. For example, fermentation of pomegranate bark residue by *A. niger* resulted in the production of citric acid **(2)** with reduced production costs, while adding value to a residue usually directed for disposal [137]. High yields (7.8 to 14.4 g/L) of dry biomass, with protein contents ranging from 33.8 to 50.8%, and ethanol were recovered from the fermentation of effluents from the wheat starch industry by the fungi *A. oryzae* and *R. oryzae*. This kind of process contributes to the development of a circular economy, because the large volume and high chemical demand of oxygen in effluents from the starch industry represent an environmental burden and an additional cost as they create a need for effluents to be treated before being discarded [138]. The filamentous fungi *Actinomucor elegans* and *Umbelopsis isabelin* were simultaneously used for the enrichment of white grape-producing bagasse, a residue with little nutritional attractiveness. The fungi were able to increase the γ-linolenic acid and carotenoid contents, improving the nutritional content of bagasse, and thus creating the possibility of reintroducing bagasse into the production chain as a low-cost functional food for humans [139].

## 6. Conclusions

Fungi and their metabolites have important industrial applications in high-value-added products and have potential for the development of nutraceuticals that can contribute to the prevention of NTCD and improve health, especially in terms of human aging. In addition, fungi are suitable in the production of natural food additives such as colorants and stabilizers that have lower health risks than synthetic food additives, and bioactive metabolites for pharmacological use such as enzymes, statins, and antitumor agents. Fungal antioxidants have applications in both food preservation and the combat of oxidative stress in the human body, with positive outcomes for several diseases such as cancer. The use of metabolic engineering techniques has facilitated the overcoming of some obstacles to explore the pharmacological potential of fungi, even those producing toxic substances such as some species of the genera *Monascus*, *Aspergillus*, and *Fusarium*. Modern approaches have been successfully utilized to evaluate the interference of additives derived from fungi with the organoleptic properties and quality of food. Strategies currently available for scaling up metabolite production include direct genetic alteration with tools such as CRISPR-Cas9 and gene recombination. Research on the use of agro-industrial byproducts for sustainable fungal fermentation has shed light on its remarkable economic importance to the production of natural additives, food, drugs, and nutraceuticals. Further in vivo antioxidant activity studies of fungal metabolites are still scarce; however, new insights are required to expand the use of metabolites from filamentous fungi to improve human health in the 21st century.

## Figures and Tables

**Table 1:** Examples of food additives and nutraceuticals from fungi.

Category	Active Component	Bioactivity	Fungal Source	References
Nutrient	Vitamin C **(55)**	Antioxidant	*Dictyophora indusiata*	[14]
Secondary Metabolites	Resveratrol	Antioxidant	*Pleurotusostreatus*	[15]
	Agmatine **(28)**	Neurological benefits	*Aspergillus oryzae*	[16]
ω-6 Polyunsaturated fatty acid	γ-linolenic acid	Anti-inflammatory	*Mucor circinelloides*	[17]
	Arachidonic acid	Development of the nervous central system and enhancement of immune response	*Mortierella alpina*	[18]
Probiotic	Whole cell	Increase of beneficial bacteria population in gastrointestinal tract	*Saccharomyces boulardii*	[19]
Nutraceutical Enzymes	Fibrino(geno)lytic enzymes	Antithrombotic	*Penicillium* sp.	[20]
			*Rhizopus microsporus*	[21]
	Lipase (Lipopan F)	Decrease glycemic response	*Rhizopus oryzae*	[22]
Fortified Nutraceuticals	Folate in fermented maize-based porridge		*Saccharomyces cerevisiae*	[23]

**Table 2:** Examples of non-coloring natural additives from fungal origin.

Function	Compound Name and Structure	Fungus	Main Uses	References
Acidulant	Malic acid 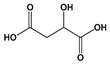	*A. niger*	Candies, soft drinks, baked goods	[44]
Antioxidant	Gallic acid 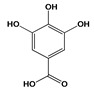	*A. niger*	Any food susceptible to oxidation	[45]
Thickener agent	Galactomannans and β-1,3-glucans 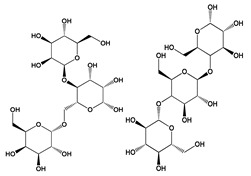	*Aspergillus terreus*	Sauces, soups, puddings, fillings	[46]
Emulsifier	Sorbitol 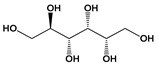	*Neopestalotiopsis* sp.	Baked goods and desserts	[47]
Flavoring agent	Dihydro-β-ionone 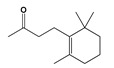	*Volvariellavolvacea*	Beverages, ice creams, candies	[48]
Preservative	Natamycin 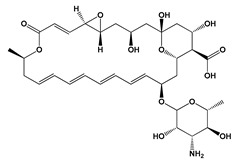	*Streptomyces gilvosporeus*	Fungicide used as biopreservative in dairy and meat products	[49]
Sweetener	Mogroside V 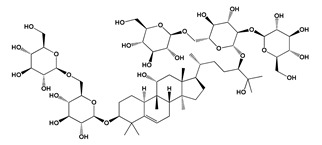	*Diaporthe angelicae* and *Fusarium solani*	Juices, soft drinks, cereals, confectionary, baked goods	[50]

**Table 3:** Cytotoxic activity of some fungal secondary metabolites against several human tumor cell lines.

Metabolite	Fungal Origin	Yield	Cytotoxic Activity				Reference
			Human Tumor Cell	IC_50_	Control	IC_50_	
7-Desmethyl-6-methylbostrycoidin **(32)**	*F. solani*	7 mg/4.3 g extract	Breast (MDA MB 231)	0.73 µM	Doxorubicin	0.07 µM	[69]
			Pancreatic (MIA PaCa2)	0.64 µM		0.04 µM	
			Cervical (HeLa)	0.71 µM		0.05 µM	
			Non small-cell lung (NCI HI975)	0.34 µM		0.03 µM	
			Lung fibroblast (WI38)	6.42 µM		0.35 µM	
7-Desmethylscorpinone **(33)**	*F. solani*	5 mg/4.3 g extract	Breast (MDA MB 231)	1.51 µM	Doxorubicin	0.07 µM	[69]
			Pancreatic (MIA PaCa2)	0.98 µM		0.04 µM	
			Cervical (HeLa)	0.96 µM		0.05 µM	
			Non small-cell lung (NCI HI975)	0.61 µM		0.03 µM	
			Lung fibroblast (WI38)	5.84 µM		0.35 µM	
Ergosterol **(43)**	*Penicillium chrysogenum*	ni	Breast (MCF-7)	0.10 mM	ni	ni	[70]
	*V.volvacea*	500 mg/725 g extract	Prostate (PC-3M)	27.98 ± 0.97 µM	5-Fluorouracil	64.35 µM	[68]
Ergosterol peroxide **(44)**	*V. volvacea*	15 mg/725 g extract	Prostate (PC-3M)	23.15 ± 1.54 µM	5-Fluorouracil	64.35 µM	[68]
3β, 5α, 9α, 14 α-Tetrahydroxy-ergosta-7,22-dien-6-one **(45)**	*V. volvacea*	5.9 mg/725 g extract	Liver (HepG2)	20.72 ± 0.76 µM	5-Fluorouracil	54.74 µM	[68]
3β, 5α, 9α-Trihydroxy-ergosta-7,22-dien-6-one **(46)**	*V. volvacea*	12.5 mg/725 g extract	Gastric (SGC-7901)	12.03 ± 0.77 µM	5-Fluorouracil	75.05 µM	[68]
			Liver (HepG2)	5.90 ± 0.44 µM		54.74 µM	
Hypocriol A **(24)**	*Hypocrea* sp.	50.3 mg/63.2 g extract	Colorectal (HCT116)	18.6 ± 0.7 µM	Cisplatin	18.8 ± 1.9 µM	[66]
			Cervical (HeLa)	7.7 ± 0.4 µM		14.7 ± 0.8 µM	
			Lung (A549)	25.3 ± 2.5 µM		13.8 ± 1.2 µM	
			Breast (MCF-7)	19.7 ± 0.4 µM		17.6 ± 2.4 µM	
Hypocriol F **(25)**	*Hypocrea* sp.	11.2 mg/63.2 g extract	Colorectal (HCT116)	2.7 ± 0.6 µM	Cisplatin	18.8 ± 1.9 µM	[66]
			Cervical (HeLa)	4.6 ± 0.1 µM		14.7 ± 0.8 µM	
			Lung (A549)	15.3 ± 1.6 µM		13.8 ± 1.2 µM	
			Breast (MCF-7)	23.6 ± 1.3 µM		17.6 ± 2.4 µM	
Terrein **(23)**	*A. terreus*	537.26 ± 23.42 g/kg extract	Colorectal (HCT-116)	12.13 µM	Doxorubicin	0.11 µM	[63]
			Hepatocellular (HepG2)	22.53 µM		0.85 µM	
Trichocarane E **(26)**	*I. fumosorosea*	30 mg/200 g extract	Breast (MDA)	0.13 µg/mL	Cisplatin	2.90 µg/mL	[67]
			Breast (MCF-7)	2.46 µg/mL		1.14 µg/mL	
			Ovary (SKOV-3)	1.01 µg/mL		3.80 µg/mL	
			Cervical (Hela)	2.32 µg/mL		2.24 µg/mL	
			Lung (A549)	1.40 µg/mL		2.13 µg/mL	
			Liver (HepG2)	1.87 µg/mL		0.62 µg/mL	
Trichocarane F **(27)**	*I. fumosorosea*	41 mg/200 g extract	Breast (MDA)	0.89 µg/mL	Cisplatin	2.90 µg/mL	[67]
			Breast (MCF-7)	4.38 µg/mL		1.14 µg/mL	
			Ovary (SKOV-3)	1.46 µg/mL		3.80 µg/mL	
			Cervical (Hela)	4.57 µg/mL		2.24 µg/mL	
			Lung (A549)	1.66 µg/mL		2.13 µg/mL	
			Liver (HepG2)	3.66 µg/mL		0.62 µg/mL	

Note: ni = not informed.

**Table 4:** Examples of non-cytotoxic biologically active fungal secondary metabolites.

Fungal Species	Bioactive Compound	Bioactivity			Health Benefit	References
		Target of Inhibitory Activity	Value	Control		
*Cladosporium sphaerospermum*	Cladosin L 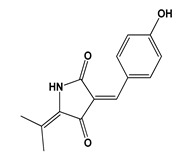	*Staphylococcus aureus*	MIC = 25–50 µM	ni	Antibacterial	[78]
*Fusarium chlamydosporum*	Chlamydosterol A 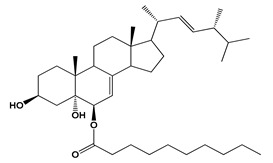	5-lipoxygenase (5-LOX)	IC_50_ = 3.06 µM	Indomethacin IC_50_ =1.13 µM	Anti-inflammatory	[79]
*Hypoxylon* sp.	Hypoxylon xanthone A 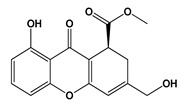	LPS-induced NO production	>70% at 1 µM	Minocycline >60% at 1 µM	Anti-neuroinflammatory	[80]
*Rhizopycnis* *vagum*	Rhizovagine A 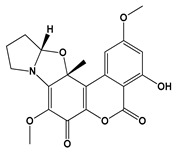	Acetylcholinesterase enzyme	IC_50_ = 43.1 µM	Tacrine hydrochloride IC_50_ = 6.1 µM	Treatment for Alzheimer’s disease	[81]
*Saccharicola* sp.	Speciosin U 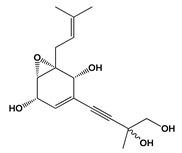	Acetylcholinesterase (huAChE-ICER)	IC_50_ = 0.037 ± 0.01 mg.mL^−1^	Galantamine IC_50_ = 0.076 ± 0.01 mg.mL^−1^	Treatment for Alzheimer’s disease	[82]
*Trichoderma* sp.	Coniothyrinone A 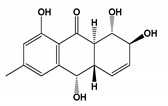	*Vibrio anguillarum*	MIC = 1.56 µM	Ciprofloxacin MIC = 0.625 µM	Antibacterial	[83]

ni = not informed.

**Table 5:** Mushroom-originated compounds and health benefits related to non-transmissible chronic diseases (NTCD).

Mushroom Species	Popular Name	Origin	Related Compounds	Health Benefits	References
*G. lucidum*	Reishi	China and Eastern Asia	Polysaccharides	Antioxidant activity related to DAF-16/FOXO activation	[85]
*H.sinensis*	Caterpillar	Tibet	Polysaccharides	Prebiotic properties related to insulin resistance and diabetes control	[87,88]
*L. edodes*	Shitake	Eastern Asia	Spermidine **(39)**	Reduction on age-dependent memory impairment	[91]
*A. bisporus*	Champignon	Eastern Europe	Lovastatin **(40)**	LDL-cholesterol lowering	[94]
*C. cibarius*	Chanterelle				[92]
*I. badia*	Bay bolete				[92]
*L. edodes*	Shitake				[92]
*C. militaris*	Caterpillar	China, Tibet	Polysaccharides	Immunomodulation improvement	[94,95]
			Cordycepin **(41)**	Total and LDL-cholesterol lowering, reduction of hyperlipidemia caused by high-fat diets	[96]
*A. subrufescens*	Sun Mushroom	Eastern North America	Polyphenols, polysaccharides	Decrease of oxidative stress, preventing diseases like cancer and inflammation	[100,101,102]
*A. cinnamomea*	Niu-Chang-Chih	Taiwan	Antcins **(42)**		[103]

**Table 6:** Bioactive compounds produced by endophytic fungi under different fermentation conditions.

Fungal Species	Host Plant	Target Compound	Health Benefit	Methodology	Target Parameters	Reference
*A.terreus*	Coconut tree	_L_-Asparaginase	Treatment of acute lymphocytic leukemia	Factorial experimental design. Increase of scale(5-l bioreactor system).	pH, temperature, inoculum concentration.	[106]
*F. solani*	*Chonemorpha fragrans*	Camptothecin	Anticancer	Box–Behnken design using one factor at a time method.	Carbon and nitrogen sources, ethanol concentration, pH, temperature, stirring speed, incubation period, precursors and elicitors.	[107]
*Penicillium bilaiae*	*Phoenix dactylifera*	Acidic protease	Increasing in food digestibility	Response surface methodology. Plackett-Burman design. Box-Behnken design.	Temperature, initial pH, carbon and nitrogen sources, metal ions, detergents and enzyme inhibitors.	[108]
*P. ostreatus*	ni	Lovastatin **(40)**	Anti-hypercholesterolemic	Response surface methodology.	Nutrients, particle size of the solid substrate, temperature, incubation time.	[109]
*Meyerozymaguilliermondii*	leaves of *Hibiscus rosa-sinensis*			One parameter at time approach.	Nutrients, pH, inoculum size, temperature, addition of metallic ions, modulators, precursors.	[110]
*A.niger*	ni	Urease	Diuretic	Response surface methodology.	Strains, incubation time, temperature, pH, biomass, inoculum size, nitrogen content and moisture.	[111]
*Spissiomycesendophytica*	*Balanophorafungosa*	Melanin	Radioprotective, thermoregulator, antitumor, and antiviral	One parameter at time approach.	Inhibitors, culture medium, temperature, pH.	[112]
*E. nigrum*	*Taxus baccata*	Taxol	Anticancer	One parameter at a time approach. Mutant strains.	Culture medium, stirring speed, temperature, incubation period, pH, medium volume, inoculum age, inoculum size, carbon source, nitrogen source, phosphorus source, gamma radiation dose.	[113]
*Alternaria brassicae*	*Huperzia serrata*	Huperzine A	Acetylcholinesterase inhibitor	Multifactorial statistical approaches. Plackett–Burman. Central composite designs.	Culture medium composition, medium volume, inoculum age, inoculum size, incubation period, ethanol addition, pH, temperature.	[114]
*F. oxysporum*	*Dioscoreazingiberensis*	Diosgenin	Anti-cancer, anti-thrombic, anti-diabetic, cardioprotective, osteoarthritis protective activity	One parameter at time approach.	Culture medium, antibiotics, temperature.	[115]
*E. nigrum*	*Terminalia arjuna*	Digoxin	Regulating the heart rhythm and strengthening heart diffusion	One parameter at time approach.	Culture medium, temperature, elicitors, incubation time, pH, medium volume, inoculum age, inoculum size, gamma irradiation mutagenesis.	[113]
*Penicillium mallochii*	A beech tree bark from Balikesir, Turkey	Orange-red pigment	Decreasing in allergic responses to synthetic pigments	One parameter at time approach.	Culture medium, pH, temperature.	[116]
*Tausonia pullulans*	*Vinca minor*	Vincamine	Improvement of cerebrovascular and cognitive disorders and reduction the effects of certain types of stroke	Protoplasts optimization.	Incubation time, temperature, modulators, protoplast inactivation method (heat, ultraviolet, microwave, sodium nitrite, and diethyl sulfate).	[117]

ni = not informed.
